# Attachment, Social Value Orientation, Sensation Seeking, and Bullying in Early Adolescence

**DOI:** 10.3389/fpsyg.2018.00239

**Published:** 2018-02-27

**Authors:** Marco Innamorati, Laura Parolin, Angela Tagini, Alessandra Santona, Andrea Bosco, Pietro De Carli, Giovanni L. Palmisano, Filippo Pergola, Diego Sarracino

**Affiliations:** ^1^Dipartimento di Scienze e Tecnologie della Formazione, Università degli Studi di Roma “Tor Vergata”, Rome, Italy; ^2^Dipartimento di Psicologia, Università degli Studi di Milano-Bicocca, Milan, Italy; ^3^Dipartimento di Scienze della Formazione, Psicologia, Comunicazione, Università degli Studi di Bari Aldo Moro, Bari, Italy; ^4^Dipartimento di Psicologia dello Sviluppo e della Socializzazione, Università degli Studi di Padova, Padova, Italy

**Keywords:** attachment, social value orientation, sensation seeking, bullying, early adolescence

## Abstract

In this study, bullying is examined in light of the “prosocial security hypothesis”— i.e., the hypothesis that insecure attachment, with temperamental dispositions such as sensation seeking, may foster individualistic, competitive value orientations and problem behaviors. A group of 375 Italian students (53% female; Mean age = 12.58, *SD* = 1.08) completed anonymous questionnaires regarding attachment security, social values, sensation seeking, and bullying behaviors. Path analysis showed that attachment to mother was negatively associated with bullying of others, both directly and through the mediating role of conservative socially oriented values, while attachment to father was directly associated with victimization. Sensation seeking predicted bullying of others and victimization both directly and through the mediating role of conservative socially oriented values. Adolescents’ gender affected how attachment moderated the relationship between sensation seeking and problem behavior.

## Introduction

A “uniform definition” of bullying suggested by the U S Centers for Disease Control and Prevention (Gladden et al., 2014) is any undesirable, repetitive aggressive behavior(s) by youth(s) who are not siblings or current dating partners, involving an observed or perceived power imbalance. The result may often involve distress at physical, social and psychological levels. This definition builds conceptually on the extensive work by Olweus (1978, 1993,2009), and implies two parties: bullying of others and being victimized. Individuals may bully and be victims of this behavior at the same time, even though individuals tend to play only one of the two roles (Cook et al., 2010).

Bullying seems to be a widespread phenomenon in teens who differ in gender, age, ethnicity, education, and socioeconomic status (Boulton et al., 2002; Scheithauer et al., 2006; Olweus, 2009; Troop-Gordon, 2017). Involvement in bullying is observed ubiquitously across continents and cultures, ranging from 7% to more than 70% both for boys girls (Carney and Merrell, 2001; Kanetsuna and Smith, 2002; Eslea et al., 2004; Kyriakides et al., 2006; Cook et al., 2010; Williams and Veeh, 2012). The significant differences are explainable with the different definitions of bullying or being exposed to bullying given by the various observers: a rough estimate of 29–46% of children involved in bullying can be considered to be reliable. Long-term consequences of being bullied seem to include serious mental disturbances (Arseneault, 2017; Evans-Lacko et al., 2017).

Theories that attribute bullying to being raised either in difficult environments or in certain types of families do not sufficiently explain this behavior. On the contrary, as many studies indicated, bullying is a complex phenomenon which depends on the interaction between temperamental factors, family environment, and social value orientation (Espelage et al., 2001; Stevens et al., 2002; Cook et al., 2010; Holt et al., 2017; Troop-Gordon, 2017). Some authors also consider community factors (Randall, 1996), peer factors (O’Connell et al., 1999), and other individual factors like temperamental, social-cognitive and emotional factors (Matthiesen and Einarsen (2004).

Only temperamental factors, on the whole, can influence conducts “before” attachment experience. Temperament can be considered the innate tendency, which should be genetically given, to react in a certain way to environmental stimuli and specifically to intensity, frequency and threshold of affective responses (Kernberg, 2005). Among temperamental factors, sensation seeking was chosen to be taken into consideration for several reasons. Hoyle et al. (2002) define it as a need for varied, novel, and complex sensations and experiences and the willingness to take physical and social risks. Several studies show that sensation seeking may be an effective predictor of a wide range of problem behaviors in adolescence (Zuckerman, 1994, 2007; Hoyle et al., 2002; Sarracino et al., 2011) and, specifically, of the likelihood of engaging in bullying and of being victims of bullying (Lovegrove et al., 2012).

Regarding family environment, previous studies have connected bullying with limited parental involvement (Flouri and Buchanan, 2003), and absence of family cohesion in boys engaged in bullying behavior (Bowers et al., 1992). A large meta-analysis by Lereya et al. (2013), conducted on studies from 1970 to 2012, shows that both victims and bully/victims are more likely to have been exposed to negative parenting behavior. The effects are generally more significant for bully/victims than for victims. Positive parenting behavior is on the contrary to be considered protective against victimization from peers. The protective effects were generally small to moderate so negative parenting behavior is related to the risk of becoming a bully/victim or victim of bullying at school.

Attachment theory constitutes a potential framework for explaining the association between relationships with parents and problematic relationships with peers. A link between insecure attachment style and problem behavior like bullying seems to be particularly noteworthy during adolescence, which is a period characterized by the reorganization of the relationship with parents, and by the gradual transfer of some attachment functions to peers (Allen and Tan, 2016). A study on 1,921 adolescents, aged 10–18 years old (Kõiv, 2012), found that adolescent victims of bullying were less securely attached than adolescents who engage in bullying and peers not involved in bullying. In the same study, bullying adolescents had higher levels on avoidant attachment scales than adolescents who were bullied and their peers not involved in bullying. Unsecure attachment can be related to the social behavior of bullying people, characterized by high aggression and poor social skills (Marini et al., 2006; Gradinger et al., 2009). Anxious and avoidant attachment were found by some authors to be typical of the victims of bullying (Ireland and Power, 2004). Anxious and avoidant maternal attachment were also positively related to interpersonal aggression in a college student sample (Cummings-Robeau et al., 2009). On the whole, it seems that individuals with secure attachments are less likely to bully others or be bullied by others (Murphy et al., 2017).

### From Attachment to Social Value Orientation: The Prosocial-Security Hypothesis

Although many studies indicated a relation between attachment insecurity and bullying, there are few studies on *how* attachment might affect bullying behaviors and being victims of bullying. Is the link between attachment and bullying direct or mediated by other factors? A fascinating hypothesis in the literature is that the experience of growing up in a nurturing and responsive family environment, and consequently attachment security, may facilitate a prosocial value orientation—i.e., relatively stable individual preferences or desirable goals that reflect socialization and that serve as guiding principles during people’s lives (Schwartz, 1992; Bilsky and Schwartz, 1994; Knafo and Schwartz, 2003). According to this *prosocial-security hypothesis*, the internalization of a secure internal model of self and others may facilitate the development of “conservative social-oriented values”—i.e., being benevolent toward others, preferring security, being tied to traditions, behaving similarly to people considered to be friends and peers. These values can be measured on specific rating scales: universalism, benevolence, tradition, conformity, and security (van Lange et al., 1997). Individuals who scored higher on these values, in particular on benevolence, remembered warmer and less aggressive parents (Aluja et al., 2005). The choice of conservative social-oriented values, in turn, may protect teens from problem behaviors, and more specifically from externalizing behaviors (Barnea et al., 1992). Moreover, high levels of benevolence were related to socialized behaviors (Aluja-Fabregat et al., 1999). On the contrary, insecure adolescents may have learned to perceive interpersonal relationships as threatening, and this may increase “dynamic self-oriented values” (including power, achievement, hedonism, stimulation, and self-direction), as shown by van Lange et al. (1997; see also Sarracino et al., 2011; Sarracino and Innamorati, 2012). People who prefer these values, especially power, remember more neglecting and less warm parents (Meesters et al., 1995). Consequently, competitive and aggressive behaviors may be favored (Aluja-Fabregat and Torrubia-Beltri, 1998) and transmitted across generations (De Carli et al., 2016, 2017a,b; Wang et al., 2017).

**FIGURE 1:**
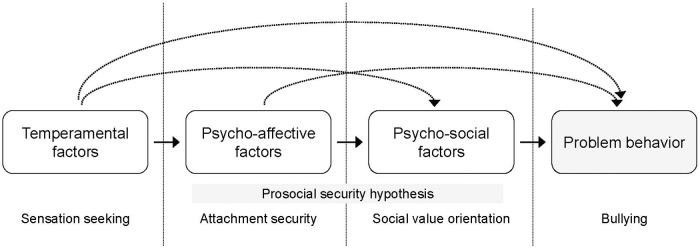
A unifying view of the relationships between temperamental, psycho-affective and psycho-social factor affecting problem behavior in adolescence.

A number of studies empirically supported the prosocial-security hypothesis through different phases of adolescence and early adulthood, showing a significant association between attachment security and prosocial values (van Lange et al., 1997; Mikulincer et al., 2003; Sarracino et al., 2011; Sarracino and Innamorati, 2012). Although we are far from having a comprehensive view, a plausible model of the relations among temperamental factors, attachment, social value orientation, and problem behaviors in adolescence can be suggested (**Figure 1**).

### The Role of Gender

Bullying concerns both males and females. Although recent studies indicate that girls are more physically aggressive than in the past (see for example, Jamal et al., 2015), the psychological repercussions on girls and boys may differ (Besag, 2006). In fact, girls are usually involved in more indirect and relational forms of bullying than boys (Crick and Grotpeter, 1995; Caravita et al., 2009; Jimerson et al., 2009). These differences are in line with evidence that boys are more likely to have externalizing behaviors than girls—with the exception of hedonistic habits like smoking and drinking (for a meta-analysis, see Byrnes et al., 1999). Moreover, boys prioritize dynamic self-oriented values (i.e., achievement and power), while girls tend to favor conservative social-oriented values (i.e., benevolence and universalism). Thus, boys are believed to be more likely to experience problematic behaviors than girls, due to their attempts to achieve social power and authority with respect to their peers (see also Wilson and Daly, 1985; Liu et al., 2007). In these studies, boys also scored higher in sensation seeking than girls.

Moreover, several studies found gender differences in adolescents’ attachment. Adolescent girls report greater attachment security with their mothers than to their fathers; conversely, while boys experience greater attachment security with their fathers (Verschueren and Marcoen, 2005; Diener et al., 2008; Del Giudice, 2009; Sarracino et al., 2011). Williams and Kennedy (2012) observed that girls were more likely to be involved in bullying when they had higher levels of attachment avoidance and attachment anxiety with their mothers, and higher levels of attachment anxiety with their fathers. On the other hand, boys were more likely to bully when they experienced higher levels of attachment anxiety with their fathers. In light of these findings, Sarracino et al. (2011) hypothesized that gender not only has a direct effect on the single variables such as attachment, social value orientation, sensation seeking and bullying, but may influence the prosocial-security model. In other words, gender may affect the way attachment mediates the relationship between sensation seeking and problem behavior.

### The Present Study

While the different studies focused on specific aspects of the problem, to date few models consider and integrate the links between temperamental, psychological and psychosocial variables, which may be involved in adolescent bullying. The contribution of the present study consists in the attempt to extend the prosocial-security hypothesis to the study of bullying. We have included sensation seeking in this model because, in a previous study, two of the authors of this paper found sensation seeking to be strongly tied to at risk behavior in pre-adolescence through the mediation of attachment (Sarracino et al., 2011), and decided to verify if the same factor is a predictor of bullying.

Specifically, our study explored the relationships between sensation seeking (as a temperamental predisposition to risk-taking), attachment security (related to family environment and parenting style), and social value orientation (connected to family and the socio-cultural context), and how this network of innate and cultural-driven influences can affect bullying-related behaviors (bullying or being bullied) in early adolescence. In this regard, we explored the possibility that a social value orientation can mediate the relationship between attachment security and both bullying and being victimized by bullying. In line with Sarracino et al.’s (2011) findings, we expected that more secure adolescents would have more “conservative social-oriented values,” less sensation seeking, and fewer bullying-related behaviors. Conversely, we hypothesized that insecure attachment, dynamic self-oriented values, and sensation seeking would be associated with more bullying-related behaviors.

Another aim of the study was to explore the role of gender in our model. Specifically, we expected different patterns for boys and girls, in which attachment moderates the relationship between sensation seeking and bullying.

## Materials and Methods

### Participants

Research assistants recruited 375 Italian early adolescents from two middle schools in Northern Italy. Average ages of participants ranged from 11 to 16 years (*M* = 12.58, *SD* = 1.08). Forty-seven percent of participants were 11–12 years old, 49% were 13–14 years old, and 4% were 15–16 years old. Fifty-three percent of participants were female. Seventy-six percent of participants reported being born in Northern Italy, 7% in Central or Southern Italy, 4% in other European countries, and 13% in non-European countries. With regard to parent nationality, 77% of parents were both Italians, 19% were both foreigners, and 4% were mixed. Eighty-one percent of participants reported living with both parents, 15% with mothers only, 2% only with their fathers, 1% with other relatives, and 1% lived in welfare structures. Twelve percent of participants reported living in a family with a low or medium-low income, 53% reported belonging to a family with a medium income, and 35% declared a medium-high or high income. Non-native Italian participants spoke and understood Italian adequately as attested by their teachers, and thus the school or parents deemed no help was necessary.

### Procedure

The present study is part of a multidisciplinary research project concerning the role of psychodynamic factors in problem behaviors in different stages of life (Innamorati et al., 2010; Dellantonio et al., 2012; Lo Verde et al., 2012; Sarracino et al., 2014; Sassaroli et al., 2015). The review board of the University of Rome “Tor Vergata” approved the study protocol, in accordance with the Helsinki Declaration. After the school principals’ approval of the study, research assistants described it to the classes, answered questions, and provided them with the informed consent form to be signed by parents, along with a letter, signed by the study authors and the school principals, explaining the importance of collaboration between schools and the universities. Participation rate was unexpectedly high (98%), which may be explained by the fact that data collection occurred during school hours, replacing the usual lessons. At a given date, research assistants administered during class information forms (containing questions on gender, age, birthplace, parents’ nationality, family situation, and income), and a set of pencil-and-paper questionnaires. The questionnaire took approximately 1 h to complete. A research assistant informed participants that the questionnaire was anonymous and that they would not be judged by their answers. Subsequently, she explained the aims of the study and answered any further questions.

### Measures

#### Attachment Security

Attachment security during early adolescence to each parent was assessed by means of the Security Scale (SS; Kerns et al., 1996). The SS is a 15-item questionnaire for children and early adolescents. Several studies showed a good validity of the Security Scale as a measure of perceived attachment security for adolescents (e.g., Kerns et al., 1996; Van Ryzin and Leve, 2012).

The Italian version of the SS (Sarracino et al., 2011) has a 4-point Likert format. An example item is: “Some kids find it easy to trust their mom.” We calculated the mean of the scores in order to obtain a single score. Reliability coefficients for the mother and father versions were α = 0.77 and α = 0.80 respectively, in line with findings reported by Kerns et al. (1996) and Sarracino et al. (2011).

#### Social Value Orientation

Social value orientation was measured using the Portraits Values Questionnaire (PVQ; Schwartz et al., 2001). This inventory consists of 40-item in terms of short verbal descriptions, which refer to ten values: Power, achievement, hedonism, stimulation, self-direction, universalism, benevolence, tradition, conformity, and security. Each item describes a third person that points to the importance of a value (e.g., “It is very important to him/her to help people around him/her. He/she wants to care for their well-being” as an example of the “self-transcendence” item). The 10-value items are rated on a 6-point Likert scale from 1 (not at all like me) to 6 (very much like me). Previous factor analyses (Hinz et al., 2005; Sarracino et al., 2011) suggested a two-factor structure, the first of which indicated a “dynamic self-orientation” (including power, achievement, hedonism, stimulation, self-direction), and the second describing a “conservative social orientation” (including universalism, benevolence, tradition, conformity, security). Several studies used the PVQ to assess value constructs of early adolescents across a wide range of cultures (Schwartz et al., 2001; Hinz et al., 2005; Liu et al., 2007; Sarracino et al., 2011). For the Italian version of the PVQ used in the present study, *α* coefficients were 0.71 for dynamic self-orientation and 0.73 for conservative social orientation. PVQ indexes were adequate and demonstrated good convergent and discriminant validity in the multitrait-multimethod (MTMM) analysis and exhibited good construct validity (Schwartz et al., 2001).

#### Sensation Seeking

The dispositional risk for various problem behaviors related to sensation seeking was assessed using the Brief Sensation-Seeking Scale (BSSS-8; Hoyle et al., 2002; Stephenson et al., 2007), which consists of 5-point scales, ranging from “Strongly disagree” to “Strongly agree” (e.g., “I like to do frightening things”). The brief version of the scale consisted of eight items, and was back translated into Italian by two professional translators. Its internal reliability was satisfactory (*α* = 0.77), and in line with findings from previous studies on adolescents (Hoyle et al., 2002; Sarracino et al., 2011). In order to validate the Italian version of the Sensation Seeking Scale we performed a Confirmatory Factor Analysis. The hypothesized one factor solution (Hoyle et al., 2002) showed an adequate fit (*χ*^2^ = 33.98, *p* = 0.02, CFI = 0.99, TLI = 0.98, RMSEA = 0.046, *p* = 0.57, 95% CI [0.02, 0.07], SRMR = 0.05). Therefore the subsequent analysis were performed using a composite score computed as the mean of the items.

#### Self-Reported Bully/Victim Behaviors

In order to assess participant self-reports of bullying behaviors, we used the Bully/Victim Questionnaire (BVQ; Olweus, 1996, 2002). The BVQ is widely used throughout the world and has satisfactory psychometric properties in terms of construct validity and reliability (e.g., Kyriakides et al., 2006). For the purposes of the present study, we selected 16 items of the questionnaire: items 5–12 for BV (i.e., the act of bullying against the child who is responding to the instrument) and items 25–32 for BO (i.e., bullying behavior manifested by the respondent; **Table 1**). For each item, adolescents indicated how often they have been bullied or how often they bullied others during the past couple of months: never or hardly ever, sometimes, or frequently. Internal consistency, as measured by Cronbach’s *α* coefficient, was 0.79 for the “Being Victimized” (BV) scale and 0.78 for the “Bullying Others” (BO) scale.

**Table 1:** Item content of the revised Olweus Bully/Victim Questionnaire used in this study.

Bullying others

Item No. Description
(1) I called another student(s) mean names, made fun of, or teased him/her in a hurtful way.
(2) I kept him/her out of things on purpose, excluded him or her from my group of friends, or completely ignored him or her.
(3) I hit, kicked, pushed, and shoved him or her around or locked him or her indoors.
(4) I spread false rumors about him/her and tried to make others dislike him/her.
(5) I took money or things from him or her or damaged his/her belongings.
(6) I threatened or forced him/her to do things he/she didn’t want to do.
(7) I bullied him/her with mean names or comments about his/her race or color.
(8) I bullied him/her with mean names, comments, or gestures with a sexual meaning.

Being victimized

Item No. Description
(1) I was called mean names, was made fun of, or teased in a hurtful way.
(2) Other students left me out of things on purpose, excluded me from their group of friends, or completely ignored me.
(3) I was hit, kicked, pushed, shoved around, or locked indoors.
(4) Other students told lies or spread rumors about me and tried to make others dislike me.
(5) I had money or things taken away from me or damaged.
(6) I was threatened or forced to do things I didn’t want to do.
(7) I was bullied with mean names or comments about my race or color.
(8) I was bullied with mean names, comments, or gestures with a sexual meaning.

### Data Analysis

We performed all the analyses with R software, using the package for structural equation modeling “lavaan” (Rosseel, 2012). In order to handle missing values (89.6% of the respondents had no missing values, with a maximum of 2.9% per variable), we imputed data using the EM algorithm (Schafer and Graham, 2002). Then we adopted a more conservative approach, and repeated all the analyses performing multiple imputations with the “Amelia” package (Honaker et al., 2011). We reported only single imputation analyses since results did not differ for multiple imputed data.

## Results

### Descriptive Analysis

**Table 2** presents the descriptive analyses of the variables of the study divided by gender. Boys showed higher scores for sensation seeking, dynamic self-oriented values (PVQ), and bullying behaviors. Girls had higher scores on conservative social-oriented values.

**Table 2:** Descriptive statistics for boys and girls.

	Boys		Girls				
	*M*	*SD*	*M*	*SD*	*t*	*df*	*p*
BSSS – Sensation seeking	3.27	0.81	2.98	0.79	3.51	366.09	0.001
SS – Attachment to mother	2.91	0.46	2.95	0.51	-0.79	367.96	0.43
SS – Attachment to father	2.84	0.55	2.73	0.59	1.82	365.37	0.07
PVQ – Dynamic self-orientation	0.12	0.50	-0.10	0.54	4.02	368.97	<0.001
PVQ – Conservative social orientation	-0.11	0.36	0.04	0.39	-3.83	368.70	<0.001
BVQ – Bullying others	1.32	0.34	1.19	0.26	4.04	328.71	<0.001
BVQ – Being victimized	1.37	0.36	1.40	0.39	-0.94	372.56	0.35

SS, Security Scale; PVQ, Portraits Value Questionnaire; BSSS, Brief Sensation Seeking Scale; BVQ, Bully/Victim Questionnaire.

### Effects of Sensation Seeking, Attachment Security, and Social Values on Bully/Victim Behaviors

In order to test the prosocial security hypothesis, we performed a path analysis, which estimated the effects of sensation seeking, attachment security, and social value orientation on the bully/victim behaviors. The developmental onset of the predictors determined the direction of the effects of the path models. Thus, since sensation seeking is a personality trait (e.g., Zuckerman, 2007), we hypothesized that it affects attachment security, which depends on environmental factors and develops later in childhood (e.g., Pasco Fearon et al., 2006). In a similar way, our path model relied on the assumption that attachment security matures prior to social value orientation and therefore influences the latter. Finally, we assumed that attachment security, social value orientation and sensation seeking affect bullying at a behavioral level. We allowed for the two attachment security factors to co-vary with each other, as well as the two social value orientation factors and the two bullying outcome variables (bullying and victim of bullying).

We used the following indices to evaluate the goodness of fit of the model: The chi-square statistic, which should be non-significant; the comparative fit index (CFI) and the Tucker-Lewis index (TLI), which should approach 0.95 to indicate optimal fit (Brown, 2015); the root mean squared error of approximation (RMSEA) and the root mean square residual (SRMR), which should approach 0.05 and 0.08, respectively, to indicate a good fit (Kline, 2015); non-significant probability values associated with the Cfit of RMSEA. The sample size seems adequate for the requirements of structural equation models presented by Kline (2015).

**FIGURE 2:**
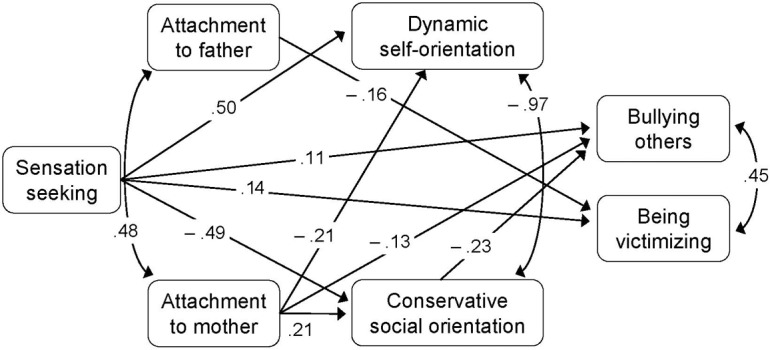
Path analysis.

**Figure 2** illustrates the path analysis of the hypothesized model. The overall fit measures indicated that the fit of the model was adequate (*χ*^2^ = 5.20, *p* = 0.07, CFI = 0.99, TLI = 0.98, RMSEA = 0.06, *p* = 0.26, 95% CI [0.00, 0.14], SRMR = 0.005). Significant paths showed the effect of sensation seeking in predicting BO and BV both directly and, only for BO, through the mediating role of conservative social-oriented values (mediated path: *b* = 0.04 [β = 0.11], *p* < 0.001, 95% CI [0.019, 0.059], total effect: *b* = 0.09 [β = 0.25], *p* < 0.001, 95% CI [0.053, 0.120]). The results also showed direct significant paths from attachment to the outcome variables.

In order to test the mediation effect of social orientation in the association between attachment to mother and bullying, we computed the indirect effect. Results show a significant mediation effect (*b* = -0.029, [β = -0.05], *z* = -2.93, *p* = 0.003, 95% CI [-0.048, -0.010]) and a significant total effect (*b* = -0.128, [*β* = -0.22], *z* = -3.88, *p* < 0.001, 95% CI [-0.192, -0.063].

### Gender Effect

**FIGURE 3:**
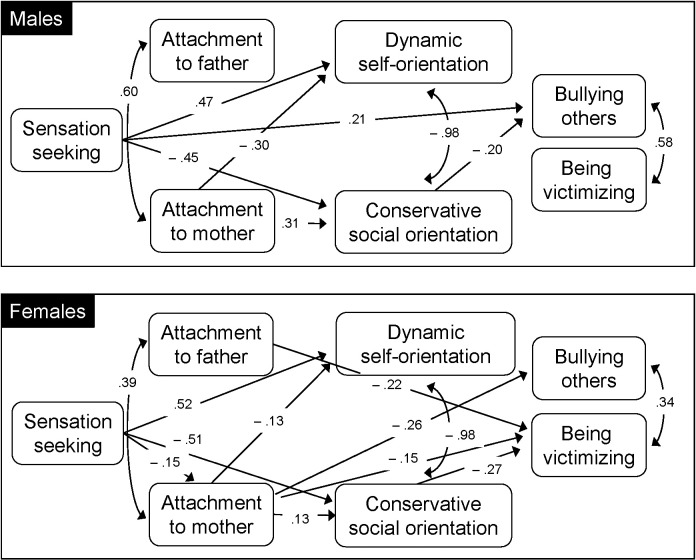
Gender effect on attachment to mother’s role as a moderator between sensation seeking and bullying of others.

In order to study whether the same pattern of associations was found both in males and females, we tested the previous model in a multiple group analysis. The sample size can support only an exploratory approach, so the findings have to be considered only preliminary and needs further replications. The fit indices can be considered adequate (*χ*^2^ = 7.06, *p* = 0.13, CFI = 0.99, TLI = 0.98, RMSEA = 0.06, *p* = 0.31, 95% CI [0.00, 0.14], SRMR = 0.005). The significant paths are presented in **Figure 3**.

We also repeated the test of the indirect effect of social orientation in the association between attachment to mother and bullying to explore whether it is present both for males and females. Results show a significant mediation effect of social orientation in males (*b* = -0.045, [β = -0.06], *z* = -2.07, *p* = 0.04, 95% CI [-0.09, -0.002]) but not in females (*b* = -0.01, [β = -0.04], *z* = -1.71, *p* = 0.09, 95% CI [-0.03, 0.002]).

## Discussion

The relationship we found between attachment security and bullying/victim behavior is consistent with prior evidence, indicating that secure adolescents tend to have higher levels of emotional and social adjustment, relationships that are more positive with both family and peers, and are less likely to engage in problem behaviors with parents and peers (Kerns et al., 1996). With respect to the “prosocial-security hypothesis” (e.g., van Lange et al., 1997; Mikulincer et al., 2003), our findings support this view as a plausible explanation of the link observed between attachment security and bullying. The path that emerged from our data suggests that attachment security is negatively related to bullying, both directly and by promoting prosocial values that become guiding principles. Secure individuals may have learned to recognize contexts and partners as safe and secure, and these grounding experiences may encourage more benevolent attitudes toward others.

Notably, our study seems to indicate that in adolescence, insecure attachment to a particular parent may be related to a specific bullying behavior. In particular, bullying was associated with insecure attachment to mothers, while being victims of bullying was associated with insecure attachment to fathers. This latter finding is in line with previous studies indicating that, in the case of peer-victimized adolescents, more emotional support from fathers was associated with lower levels of emotional and behavioral problems concurrently and across time (Yeung and Leadbearer, 2010). More research is needed to better understand the relationship of attachment to fathers with specific forms of bullying and other problem behaviors, in light of the results of other studies (e.g., Sarracino et al., 2011; Williams and Kennedy, 2012).

In contrast to the direct effect of attachment to fathers on being victimized by bullies, the effect of attachment to mothers was both direct and mediated by a more conservative social orientation. This is more in line with the prosocial security hypothesis, indicating that the “mediational” role of social value orientation may be particularly important in the transition from attachment to mother to relationships with peers. Mothers are usually the main caregiving figures and have a fundamental role in the formation of adolescents’ internal representations of self and others. This not only influences the quality of the relationships with peers, but it is also likely to be associated with how adolescents process social relationships with “others”—that is, their social value orientation and the sense of belonging to a community (Mikulincer et al., 2003; Sarracino and Innamorati, 2012). Further studies are needed to investigate how the general, abstract adolescent internal model of “others” (i.e., their social value orientation) interacts with the internal model of their parents and peers (i.e., their attachment to significant figures).

Further, our study indicates that sensation seeking is an important temperamental variable during adolescence, which may be related to bullying/victim behaviors both directly and through the mediating role of conservative social-oriented values. This is consistent with previous results found by Sarracino et al. (2011), and suggests that sensation seeking should be included in the original prosocial-hypothesis model in predicting problem behavior in adolescence. In light of previous literature, it is not surprising that we found a positive association between sensation seeking and dynamic self-oriented values. Interventions to reduce problem behaviors among adolescents should consider this relation to a greater extent. In fact, a previous study found individual differences in value priorities to be related to prosocial, antisocial, and offending/delinquent behaviors (Bardi and Schwartz, 2003). Schwartz et al. (2001) suggested that when a value has a higher priority, individuals tend to overly pursue it. Furthermore, Liu et al. (2007) showed a relation between value orientation and actual behavior among early adolescents. Our results are also in line with previous findings showing a strong association between stimulation-related/hedonistic values and externalizing behavioral problems, like substance abuse, deviant and at-risk sexual behaviors (Zuckerman, 1994; Goff and Goddard, 1999; Hoyle et al., 2002; Liu et al., 2007). It is possible that individuals who seek self-enhancement tend to very strongly pursue success and dominate others. This behavioral pattern may be strengthened by perceiving interpersonal situations as potentially risky and perilous (i.e., insecure attachment working models), thus further reinforcing their “dynamic self-orientation.”

Regarding gender, in our study we found significant differences in bullying behaviors. As expected, boys scored higher than girls, in line with the greater tendency for direct aggressiveness in boys (Byrnes et al., 1999). These gender differences for bullying should only be considered in light of the other variables examined in this study. Gender also seems to play a significant role in relation to attachment to mother and father (Del Giudice, 2009), as well as preferences for specific social values. Our study is also in line with previous findings across a wide range of cultures (e.g., Schwartz and Rubel, 2005; Liu et al., 2007; Sarracino et al., 2011), which indicated that boys prioritize dynamic self-oriented values (i.e., achievement and power), while girls tend to favor conservative social-oriented values (i.e., benevolence and universalism). Thus, boys are believed to be more likely to experience problematic behaviors than girls, due to their attempts to achieve social power and authority with respect to their peers (see also Liu et al., 2007; Wilson and Daly, 1985). Boys also scored higher in sensation seeking than girls, and this may explain why they seem to be generally more exposed to problem behaviors like bullying.

Gender also affected the way in which attachment mediated the relationship between sensation seeking and bullying. Most notably, results showed a significant mediation effect of social orientation in males but not in females. This finding is both in line with the gender socialization theory, according to which it is easier for same-gender parent–child dyads to establish synchronous interactions from infancy onward (e.g., Feldman, 2003), and with previous studies, indicating that, over time, adolescent girls report feeling more distant, uncomfortable, and withdrawn from their fathers (Youniss and Smollar, 1985).

Overall, our findings confirm that gender represents a key factor in the transition from middle childhood to adolescence, and that gender differences should be expected for many aspects of adolescents’ attachment and psychosocial functioning.

### Limitations of the Study and Suggestions for Future Research

Our study has a number of limitations. Firstly, the study only employed self-report measures. The reliance on a single informant may have caused shared-method variance (i.e., the relation between variables may be in part due to similar measurement methods). Furthermore, self-report measures may cause systematic distortions when investigating problem behavior in adolescents. In fact, even though we assured anonymity to participants, their answers may not be entirely reliable, since we asked them to report if they had participated in deviant and illegal activities at times. Therefore, future research should integrate reports from caregivers and/or observational data relative to caregiver–adolescent interactions.

Second, this study has to be considered as explorative and thus causal interpretations should be made with caution since our data are cross-sectional, and the variables of the study have not been extensively researched in the early adolescence age-group. Longitudinal studies are therefore required in order to explore the direction of effects, and to understand how attachment security and other social experiences interact, and influence children’s social development in general, and bullying more specifically.

Third, our sample was somewhat homogenous concerning social class, family structure, and ethnicity. Therefore, future research should be extended to adolescents from other ethnic backgrounds, family structures (e.g., both single and dual-earners), social contexts (e.g., at-risk populations), and different nationalities.

## Conclusion

Despite its limits, this study contributes to the exploration of how early adolescents’ value orientations and bullying behaviors may be related to attachment patterns. Our findings underscore the role of attachment to parents, its association with social values and the dispositional inclinations of early adolescents. Our results further support the general hypothesis that secure attachment relationships, and the resulting emotional connectedness and support, facilitate the development of solid value structures.

Our findings also have repercussions for interventions that aim to reduce bullying and prevent short and long-term effects on mental health as well as the psychosocial adaptation of adolescents involved in bullying. Implementing psycho-educational interventions aimed at promoting secure parental relationships (e.g., Wright and Edginton, 2016) and prosocial values may prove to be an effective method for decreasing bullying, particularly during the “sensitive period” for change which characterizes early adolescence.

## Author Contributions

MI: original idea of the work, bibliographic research, introduction, and final revision. DS: first draft of the text after introduction, general supervision, design of figures, original idea of the research on attachment, and social value orientation. Other authors: data collecting and statistics.

## Conflict of Interest Statement

The authors declare that the research was conducted in the absence of any commercial or financial relationships that could be construed as a potential conflict of interest. The handling Editor declared a shared affiliation, though no other collaboration with several of the authors LP, AT, AS, PDC, and DS.
